# An orthotopic xenograft model of human nonseminomatous germ cell tumour

**DOI:** 10.1054/bjoc.2001.1884

**Published:** 2001-08

**Authors:** M L Douglas, K J Boucaut, T M Antalis, C Higgins, M F Pera, M A Stuttgen, D L Nicol

**Affiliations:** Department of Surgery, University of Queensland; Anatomical Pathology Division, Queensland Health Pathology Service, Princess Alexandra Hospital, Brisbane, Queensland, 4102; Cellular Oncology Laboratory, University of Queensland Joint Oncology Program and Queensland Institute of Medical Research, Brisbane, Queensland, 4029; Monash Institute of Reproduction and Development, Monash Medical Centre, Clayton, Victoria, 3168, Australia

**Keywords:** testicular germ cell tumour, NSGCT, metastasis, SCID, orthotopic xenograft

## Abstract

We have established the first example of an orthotopic xenograft model of human nonseminomatous germ cell tumour (NSGCT). This reproducible model exhibits many clinically relevant features including metastases to the retroperitoneal lymph nodes and lungs, making it an ideal tool for research into the development and progression of testicular germ cell tumours. © 2001 Cancer Research Campaign http://www.bjcancer.com


*British Journal of Cancer* (2001) **85**(4), 608–611
© 2001 Cancer Research Campaign
doi: 10.1054/ bjoc.2001.1884, available online at http://www.idealibrary.com on IDEAL

http://www.bjcancer.com

### An orthotopic xenograft model of human nonseminomatous germ cell tumour

**ML Douglas[1], KJ Boucaut[3], TM Antalis[3], C Higgins[2], MF Pera[4], MA Stuttgen[3] and DL Nicol[1]**

[1]Department of Surgery, University of Queensland and [2]Anatomical Pathology Division, Queensland Health Pathology Service, Princess Alexandra Hospital, Brisbane, Queensland 4102; [3]Cellular Oncology Laboratory, University of Queensland Joint Oncology Program and Queensland Institute of Medical Research, Brisbane, Queensland 4029; [4]Monash Institute of Reproduction and Development, Monash Medical Centre, Clayton, Victoria 3168, Australia

**Summary** We have established the first example of an orthotopic xenograft model of human nonseminomatous germ cell tumour (NSGCT). This reproducible model exhibits many clinically relevant features including metastases to the retroperitoneal lymph nodes and lungs, making it an ideal tool for research into the development and progression of testicular germ cell tumours. © 2001 Cancer Research Campaign http://www.bjcancer.com

**Keywords**: testicular germ cell tumour; NSGCT; metastasis; SCID; orthotopic xenograft

Non-seminomatous germ cell tumours (NSGCTs) represent approximately 40% of all testicular germ cell tumours that occur in the adult testis. These tumours are the most commonly diagnosed malignancies in young men and account for up to 1 in 7 deaths in males aged between 15 and 45 years (Bergstrom et al, 1996). Although these tumours are readily resected and, unlike other solid malignancies, respond well to adjuvant therapies up to one-third of patients with advanced disease fail to respond to treatment and consequently die from these tumours (Toner and Motzer, 1998; Benedetto, 1999).

To date, no reproducible model for NSGCTs has been described. This has been identified as a potential obstacle to further investigations of the underlying pathogenesis of these tumours (Looijenga and Oosterhuis, 1999). Here, we report the establishment of an orthotopic xenograft model of human NSGCT in mouse testis that successfully reproduces many of the elements observed in the human disease. The clinical relevance of this model makes it suitable not only for research into the underlying biomolecular processes of this disease but also in further refining the current strategies used in treating patients diagnosed with NSGCT.

### MATERIALS AND METHODS

### Human NSGCT cell culture

The NTera-2 clone 13 cell line (Thompson et al, 1984) was cloned from the original Tera-2 cell line initially derived from the pulmonary metastasis of a 22-year-old male Caucasian patient (Fogh et al, 1977). The GCT 27 C-4 embryonal carcinoma cell line was isolated from a malignant primary testicular teratoma (Pera et al, 1987). Both cell lines were maintained in a mixture of MEM-alpha medium and HAMS F12 1:1 supplemented with 10% fetal bovine serum, 50 μg $mL^{-1}$ penicillin, 100 μg $mL^{-1}$ streptomycin, 2.5% HEPES with 15 mL $L^{-1}$ 0.833% sodium hydroxide.

*Received 19 July 2000*
*Revised 2 April 2001*
*Accepted 4 April 2001*

*Correspondence to:* D Nicol

### Experimental animals

Male SCID mice were purchased at 6–7 weeks of age from the Walter and Eliza Hall Institute Breeding Facility, Melbourne, Australia and housed in a temperature-controlled, specific pathogen-free room (23°C, 12h light–dark regime) with free access to water and a standard diet (Norco, Lismore, NSW, Australia). Tumour cells were implanted when the mice were 8 weeks of age. The study was approved by the Group 5 Animal Experimental Ethics Committee of the University of Queensland and performed according to NHMRC Guidelines (AEEC Approval Number SURG/394/98, SURG/569/99).

### Xenograft model

NSGCT tumour cells were implanted by injection in the exteriorized left testis of anaesthetized mice ($1 \times 10^6$ viable cells in 0.25 mL PBS). Mice were culled when the apparent size of the tumour approached 1 $cm^3$. The testes, viscera, retroperitoneal lymph nodes, heart, lungs and brain were harvested, weighed and preserved for histological analysis. Tumour burden was defined as the weight of the tumour-affected testis expressed as a percentage of the animal's total body weight.

### Histology

Tissues taken for histological study were fixed in 10% buffered formalin for a minimum of 24 hours. The organs were then embedded in paraffin and sectioned. Sections were routinely processed and stained with haematoxylin and eosin. 4 μm thick sections were immunostained with antibodies against CD30 (DAKO, 1/30 dilution), S100 (DAKO, 1/4000 dilution), GFAP (DAKO, 1/7 dilution), AE1/AE3 (Roche, 1/600 dilution), CAM 5.2 (Becton Dickinson, 1/20 dilution) and PLAP (DAKO, 1/100 dilution). The peroxidase-antiperoxidase method was used to detect specific antibody binding.

### RESULTS

### Xenograft model of human NSGCT

The requirements for a successful model were met by implanting $1 \times 10^6$ viable NTera-2 C-13 cells in the left testis of male SCID mice. Tumours developed in all mice and approached the 1 $cm^3$ size end point (less than 5% of host animal's normal total body weight) within 4 weeks. Post-mortem examination of these mice revealed the presence of metastases in sites consistent with NSGCT progression in human patients (see below and Discussion). Repeated use of this model has demonstrated the reproducibility of this technique (100% occurrence of primary tumours, mean tumour burden at 4 weeks: 3.68 ± 0.23%, $n$ = 20).

The GCT 27 C-4 cell line did not prove to be as suitable for establishment of an animal model of human NSGCT. Primary tumours developed over a longer time period (6–7 weeks) and were less consistent in size and appearance, although the pattern of metastasis was identical to that observed with the NTera-2 C-13 cell line.

### Physical features of the NTera-2 C-13 model of NSGCT

During the 4-week period of tumour development, the mice exhibited no symptoms or signs of clinical illness apart from a visible or palpable lump in the lower abdomen or scrotum. The rapid growth of the tumours was externally visible, with lumps apparent in the scrotum and lower perineum. As these tumours increased in size, they generally moved higher into the abdomen and post mortem, were located in the groin adjacent to the bladder.

Macroscopically, the primary tumours were large, ovoid and mobile in the lower abdomen, involving the entire testis (Figure 1). The tumours remained discrete without any evidence of malignant adhesion to adjacent structures. There was no visible evidence of ascites or seeding of tumour cells in the abdomen and no obvious nodules in the viscera or lungs. Musculoskeletal structures appeared macroscopically normal. Visible secondary tumours were evident in the retroperitoneal lymph nodes in the left renal hilar lymph region.

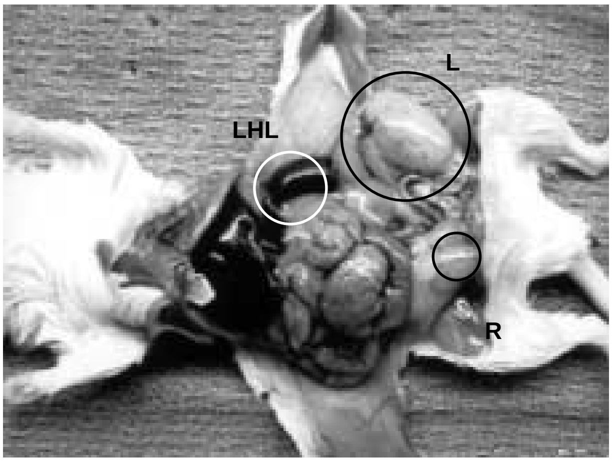

**Figure 1** Typical macroscopic appearance of a male SCID mouse 4 weeks after inoculation of the left testis with $1 \times 10^6$ viable NTera-2 C-13 cells. The tumour-affected left testis (LT) and contralateral normal testis (RT) are shown circled in black. The left renal hilar lymphatic tissue (LHL) is shown circled in white

### Histology of the NTera-2 C-13 model of NSGCT

Histological examination of the testicular tumours showed a poorly differentiated malignancy composed of sheets of tumour cells surrounding intact seminiferous tubules (Figure 2A). The cells had large vesicular nuclei with clumped chromatin, one or more large nucleoli, and indistinct cytoplasm. There was marked overlapping of nuclei and cell crowding. A high mitotic and apoptotic rate was identified. In other areas there was evidence of neuroepithelial differentiation with obvious rosette formation. Focally the tumour cells were arranged in an eosinophilic fibrillary background resembling glial differentiation. Immunoperoxidase stains revealed focal positive staining for CD30 in the large cell areas with patchy weak staining for CAM 5.2 (data not shown). The background glial areas were positive for S100 and GFAP and there was no positive staining for PLAP (data not shown).

These features are consistent with human NSGCT, with diagnostic characteristics of both embryonal carcinoma and immature teratoma (teratocarcinoma). Histological examination of other organs revealed deposits of histologically identical tumour in numerous sites in all animals. Tumour was identified in liver (Figure 2B) and lung (Figure 2C). Retroperitoneal soft tissue deposits were evident and focally these abutted the capsule of the kidney. Tumour was seen clearly within an endothelial lined lymphovascular space within the retroperitoneal tissues (Figure 2D). Metastatic deposits were also identified within the adrenal cortex and pancreas (data not shown). No metastases were identified within the kidney itself or within heart, brain or spleen.

### DISCUSSION

In the past, tumour models were based on tumours that developed spontaneously in certain species, or as a result of specific genetic abnormalities that arose either from breeding programmes or the deliberate alteration of certain genes (transgenic or 'knock-out' lines). Although valuable in studies of developmental processes, these models have not proved as useful for cancer research. Testicular germ cell tumours are particularly difficult to study using these approaches as human tumours differ markedly from spontaneous testicular tumours observed in mice (Walt et al, 1993).

Xenograft models using single-cell suspensions of tumour cells provide a distinct advantage in that cells may be studied and manipulated in vitro, complementing research performed in vivo. A number of studies have used heterotopic xenografts (most often generated by implanting tumour tissue or cells subcutaneously) to study the growth rates of tumours, the efficacy of therapeutic compounds and/or the biological properties of the tumour in vivo. Whilst these tumours are seen to grow readily and are easily monitored, these models rarely exhibit clinically important features such as metastasis. Xenograft tumours are more likely to develop in a clinically relevant way and metastasize when orthotopically implanted, where environmental conditions more accurately reflect many of the tumour–host cell interactions that take place during naturally occurring tumour development and progression (Fidler, 1990). Thus, the use of orthotopic xenografts is critical in generating clinically representative models of human disease.

Recently, Konaka et al (1999) described the first orthotopic xenograft model of human testicular germ cell tumour, where wedges of heterotopically xenografted human seminoma were implanted orthotopically in the testes of SCID mice. This model

© 2001 Cancer Research Campaign

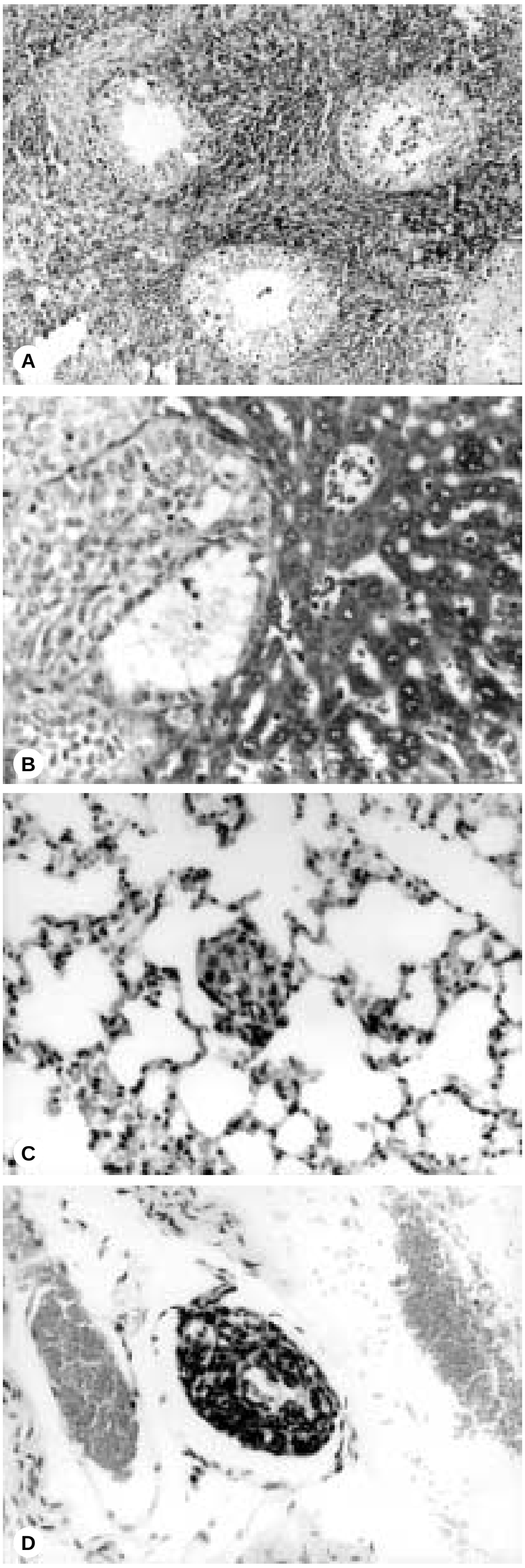

**Figure 2** (**A**) Malignant tumour surrounding native seminiferous tubules (original magnification × 100). (**B**) Tumour deposit within the hepatic parenchyma (original magnification × 250). (**C**) Pulmonary deposit of tumour (original magnification × 250). (**D**) Lymphovascular space involvement by tumour (original magnification × 250)

produced clinically relevant metastases and therefore represents a significant advance in the study of seminomatous tumours. However, there are some limitations to its usefulness in certain experimental circumstances and it may not be applicable for studying the more heterogeneous NSGCT.

We have developed a versatile orthotopic xenograft model where NSGCTs develop from cell lines rather than from grafted tumour tissue. The use of cell lines provides a number of distinct advantages. Whereas a clinical specimen from a human patient can only be used to establish a model once, cell lines allow repeated experiments using a reproducible model. The ability to culture and store cells for extended periods of time allows greater flexibility in experimental planning and experiments are not dependent on the availability of suitable patient tissue. Further, the use of cell lines obviates the necessity to maintain tumours by repeated subcutaneous implantation from mouse to mouse. An added benefit is that implantation of tumour cells by injection of single cell suspension is an easy technique to perform, increasing the reproducibility of the model and its application. More importantly, cell lines can be manipulated in vitro, allowing a wide range of investigations to be carried out, including research into the biology of particular genes. Some have argued that injections of cell suspensions do not result in as extensive metastasis as implantation of whole tumour fragments (An et al, 1999; Hoffman, 1999). Our experience with both the NSGCT model described here and a similar model of human renal cell carcinoma (RCC) also established in our laboratory (Hii et al, 1998) does not agree. In both cases, the rate of development and the pattern and extent of metastasis has been comparable if not superior to fragment implantation models of similar tumours. To compare, the seminoma model described by Konaka et al demonstrated obvious primary tumours and lymph node metastasis 11 weeks after implantation of tumour fragments, but pulmonary and visceral metastasis was not histologically evident. Our NSGCT model, using cell suspensions, repeatedly exhibits large primary tumours and lymph node metastases and histologically visible metastases in the lungs and various other organs. Similarly, our RCC model using cell suspensions yields large primary tumours and macroscopically visible pulmonary metastases within 35 days, whereas the tumour fragment model described by An et al, was reported to require 40 days. We therefore maintain that use of single cell suspensions offers many advantages and does not necessarily reduce the efficacy or relevance of an orthotopic xenograft model.

In summary, we have established an orthotopic xenograft model of human NSGCT that is easily performed, reproducible and able to generate rapid results that are clinically relevant. The primary tumours bear many characteristics typical of NSGCT as seen in human patients and are histopathologically recognizable as typical NSGCTs. To the authors' knowledge, this model is the first of its kind to be described and will be invaluable for future studies of factors that influence the development of human NSGCT as well as the development of new therapeutic strategies.

### ACKNOWLEDGEMENTS

The authors wish to thank Mr David Wiseman and the staff of the Biological Research Facility for their advice with the animal work. This research was supported by the Princess Alexandra Hospital Research and Development Foundation and the Australian Urological Foundation.

 © 2001 Cancer Research Campaign

### REFERENCES

An Z, Jiang P, Wang X, Moossa AR and Hoffman RM (1999) Development of a high metastatic orthotopic model of human renal cell carcinoma in nude mice: benefits of fragment implantation compared to cell-suspension injection. *Clin Exp Metastasis* **17**: 265–270

Benedetto P (1999) Chemotherapy of Testis Cancer. *Cancer Control* **6**: 549–559

Bergstrom R, Adami HO, Mohner M, Zatonski W, Storm H, Ekbom A, Tretli S, Teppo L, Akre O and Hakulinen T (1996) Increase in testicular cancer incidence in six European countries: a birth cohort phenomenon. *J Natl Cancer Inst* **88**: 727–733

Fidler IJ (1990) Critical factors in the biology of human cancer metastasis: twenty-eighth G.H.A. Clowes memorial award lecture. *Cancer Res* **50**: 6130–6138

Fogh J, Wright WC and Loveless JD (1977) Absence of HeLa cell contamination in 169 cell lines derived from human tumors. *J Natl Cancer Inst* **58**: 209–214

Hii SI, Nicol DL, Gotley DC, Thompson LC, Green MK and Jonsson JR (1998) Captopril inhibits tumour growth in a xenograft model of human renal cell carcinoma. *Br J Cancer* **77**: 880–883

Hoffman RM (1999) Orthotopic metastatic mouse models for anticancer drug discovery and evaluation: a bridge to the clinic. *Invest New Drugs* **17**: 343–359

Looijenga LH and Oosterhuis JW (1999) Pathogenesis of testicular germ cell tumours. *Rev Reprod* **4**: 90–100

Pera MF, Blasco Lafita MJ and Mills J (1987) Cultured stem-cells from human testicular teratomas: the nature of human embryonal carcinoma, and its comparison with two types of yolk-sac carcinoma. *Int J Cancer* **40**: 334–343

Thompson S, Stern PL, Webb M, Walsh FS, Engstrom W, Evans EP, Shi WK, Hopkins B and Graham CF (1984) Cloned human teratoma cells differentiate into neuron-like cells and other cell types in retinoic acid. *J Cell Sci* **72**: 37–64

Toner GC and Motzer RJ (1998) Poor prognosis germ cell tumors: current status and future directions. *Semin Oncol* **25**: 194–202

Walt H, Oosterhuis JW and Stevens LC (1993) Experimental testicular germ cell tumorigenesis in mouse strains with and without spontaneous tumours differs from development of germ cell tumours of the adult human testis. *Int J Androl* **16**: 267–271

*© 2001 Cancer Research Campaign*
